# Association between oligo-residual disease and patterns of failure during EGFR-TKI treatment in *EGFR*-mutated non-small cell lung cancer: a retrospective study

**DOI:** 10.1186/s12885-021-08983-2

**Published:** 2021-11-19

**Authors:** Taichi Miyawaki, Hirotsugu Kenmotsu, Hiroaki Kodama, Naoya Nishioka, Eriko Miyawaki, Nobuaki Mamesaya, Haruki Kobayashi, Shota Omori, Ryo Ko, Kazushige Wakuda, Akira Ono, Tateaki Naito, Haruyasu Murakami, Keita Mori, Hideyuki Harada, Masahiro Endo, Kazuhisa Takahashi, Toshiaki Takahashi

**Affiliations:** 1 Division of Thoracic Oncology, Shizuoka Cancer Center, 1007 Shimonagakubo, Nagaizumi-cho, Sunto-gun, Shizuoka, 411-8777 Japan; 2 Department of Respiratory Medicine, Juntendo University Graduate School of Medicine, Tokyo, Japan; 3 Clinical Research Center, Shizuoka Cancer Center, Shizuoka, Japan; 4 Radiation and Proton Therapy Center, Shizuoka Cancer Center, Shizuoka, Japan; 5 Division of Diagnostic Radiology, Shizuoka Cancer Center, Shizuoka, Japan

**Keywords:** Non-small-cell lung cancer, Oligo-residual disease, Failure pattern, EGFR-TKI, Osimertinib

## Abstract

**Background:**

Local ablative therapy (LAT) may be beneficial for patients with epidermal growth factor receptor (*EGFR*) mutated non-small cell lung cancer (NSCLC) with oligo-residual disease after treatment with EGFR tyrosine kinase inhibitor (EGFR-TKI). However, this has not been fully established. This study aimed to evaluate the predominant progressive disease (PD) pattern limited to residual sites of disease after treatment with EGFR-TKI.

**Methods:**

Patients with advanced *EGFR*-mutated NSCLC treated with EGFR-TKIs as first-line therapy were retrospectively analysed during a 7-year period. Oligo-residual disease was defined as the presence of 1 – 4 lesions (including the primary site) at 3 months from the start of EGFR-TKI treatment. The predictive factors of PD patterns after EGFR-TKI treatment were evaluated.

**Results:**

A total of 207 patients were included. Three months after the start of EGFR-TKI treatment, 66 patients (32%) had oligo-residual disease. A total of 191 patients had PD, 60 with oligo-residual disease and 131 with non-oligo-residual disease. Regarding the pattern, 44 patients (73%) with oligo-residual disease and 37 patients (28%) with non-oligo-residual disease had PD limited to the residual sites. Multivariate logistic regression analysis at 3 months from the start of EGFR-TKI treatment revealed that oligo-residual disease (*P* < 0.001), the lack of residual central nervous system metastases (*P* = 0.032), and initial treatment with osimertinib (*P* = 0.028) were independent predictors of PD limited to residual disease sites.

**Conclusions:**

This study provided a rationale for LAT to all sites of residual disease in patients with oligo-residual disease during EGFR-TKI treatment.

**Supplementary Information:**

The online version contains supplementary material available at 10.1186/s12885-021-08983-2.

## Background

Epidermal growth factor receptor-tyrosine kinase inhibitors (EGFR-TKIs) have demonstrated clinical activity in the treatment of patients with *EGFR*-mutated non-small-cell lung cancer (NSCLC) [1], Several clinical trials have indicated the superiority of EGFR-TKIs over conventional chemotherapy in terms of treatment efficacy, progression-free survival (PFS), and objective response rate in patients with *EGFR*-mutated NSCLC [1–3]. Furthermore, osimertinib, a third-generation EGFR-TKI, was associated with prolonged PFS and overall survival (OS) compared to first-generation EGFR-TKIs, including erlotinib or gefitinib, in a phase III trial [4, 5]. However, the development of resistance to EGFR-TKIs remains an obstacle to achieving disease control and long-term survival [6].

Adding local ablative therapy (LAT) to standard systemic therapy could be a treatment option that provides high local control of residual disease in patients with oligometastatic NSCLC [7–9]. The European Society for Medical Oncology defines oligometastatic NSCLC as the presence of 1 – 3 metastases and recommends the addition of LAT in these patients [10]. Previous studies have demonstrated that LAT of all sites of disease provided potential benefits in patients with synchronous oligometastatic NSCLC [11–13].

Progressive disease (PD) after first-line chemotherapy has been shown to be predominantly limited to the original site of disease, providing a rationale for the addition of LAT in patients with oligometastatic NSCLC [14, 15]. In patients with *EGFR*-mutated NSCLC, almost half had PD limited to the original sites of disease after first-line treatment with EGFR-TKIs [16–18]. No significant association was found between synchronous oligometastatic disease and patterns of initial PD after EGFR-TKI treatment [18]. However, several studies have shown that LAT to all sites of residual disease demonstrated favourable PFS and OS in patients with oligo-residual disease [19–21].

The clinical features of oligo-residual disease are unknown. Furthermore, the patterns of initial PD after first-line treatment with EGFR-TKIs remain unclear in patients with oligo-residual disease. Our study aimed to evaluate the clinical impact of oligo-residual disease on patterns of PD after EGFR-TKI treatment and establish a rationale for adding LAT to all sites of oligo-residual disease.

## Methods

### Patients

The medical records of patients with stage IV *EGFR*-mutated NSCLC who received EGFR-TKI first-line monotherapy at the Shizuoka Cancer Centre between January 2013 and December 2019 were retrospectively reviewed. This study’s protocol was approved by our institutional ethics review board (approval number: J2020-177-2020-1) and was conducted in accordance with the Declaration of Helsinki. The need for informed consent was waived due to the retrospective nature of the study.

**Fig. 1:**
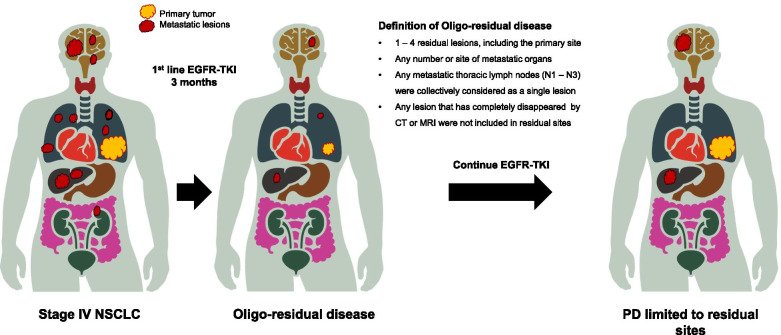
Oligo-residual disease and PD limited to residual sites. The criteria and legends for Oligo-residual disease. EGFR, epidermal growth factor receptor; PD, progressive disease; TKI, tyrosine kinase inhibitor

The collected data included age, sex, Eastern Cooperative Oncology Group performance status (ECOG-PS), smoking history, *EGFR* mutation status, metastatic details (sites, number of organs, number of metastases), and type of EGFR-TKIs. *EGFR* gene mutations were evaluated using polymerase chain reaction amplification using commercially available assays. Age, ECOG-PS, metastatic details, and the number of residual lesions were collected at 3 months ±6 weeks after the start of EGFR-TKI therapy. Two previous studies have shown that the median time from EGFR-TKI treatment initiation to maximum response was approximately 3 months [16, 22]. Therefore, we evaluated the number of patients with residual disease at 3 months after EGFR-TKI treatment initiation, to evaluate the incidence of oligo-residual disease. Synchronous oligometastatic disease was defined as the presence of 1 – 3 metastases (1 – 4 lesions including the primary site) at the time of NSCLC diagnosis [12, 14, 23]. Oligo-residual disease was defined as the presence of 1 – 4 residual lesions, including the primary site, at 3 months after the start of EGFR-TKI (Fig. 1) [19, 23]. Residual disease was defined as detectable lesions on imaging evaluation after 3 months of EGFR-TKI therapy; moreover, lesions that had completely disappeared on computed tomography (CT) or magnetic resonance imaging (MRI) were not included in the definition of residual disease. Lesions that had previously been treated with local therapies, such as radiotherapy, were excluded from the definition of “residual disease” unless they had worsened. In all analysed patients, the residual lesions were independently evaluated by a thoracic oncologist and radiologist. Additionally, patients who received LAT before EGFR-TKI treatment were excluded from this study. Furthermore, no patient who developed oligo-residual disease after EGFR-TKI therapy induction underwent LAT.

**Fig. 2:**
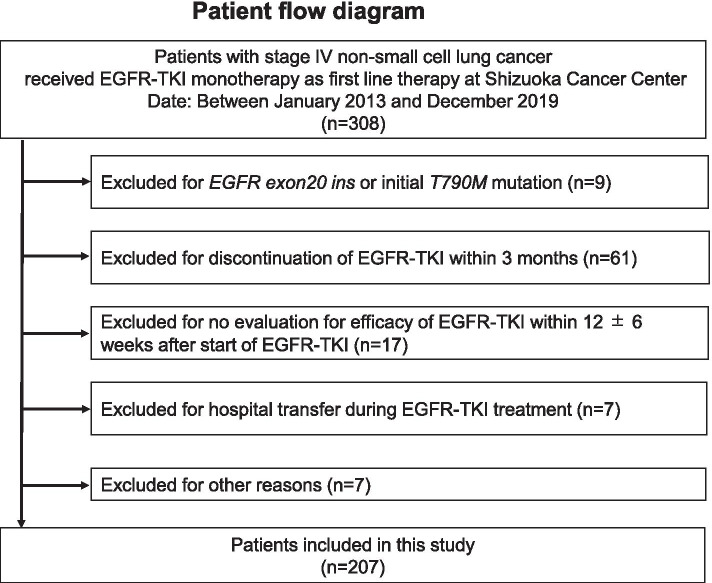
Flowchart of included and excluded patients. EGFR, epidermal growth factor receptor; NSCLC, non-small cell lung cancer; TKI, tyrosine kinase inhibitor

Among the 308 eligible patients, those with *EGFR* exon20 insertion or initial T790M mutation (*n* = 9), those who did not continue EGFR-TKI therapy for 3 months (*n* = 61, 42 due to disease progression, and 19 due to side effects), those without an efficacy evaluation at the previously mentioned time frame (*n* = 17), those transferred to other hospitals during the treatment period (*n* = 7) and other reasons (n = 7) were all excluded from this study (Fig. 2).

### Treatment and assessments

The baseline disease stage was assessed using systemic and brain imaging. Metastatic lesions were independently evaluated by a thoracic oncologist and radiologist in all patients. Based on a randomised phase II study, any metastatic thoracic lymph nodes (N1 – N3), including those in the supraclavicular fossae, were collectively considered as a single lesion [23]. PD was identified by reviewing follow-up radiological imaging, including CT and MRI, after the initiation of EGFR-TKI. Tumour responses were classified according to RECIST version 1.1 [24]. In most patients, chest and abdominal CT were performed every 6 to 12 weeks, and brain MRI was performed at the physician’s discretion. PD patterns were classified as PD limited to residual sites of disease, PD at new sites, and PD at both sites (Fig. 1).

### Statistical analysis

PFS was calculated from the residual disease evaluation to the first evidence of disease progression or death from any cause and estimated using the Kaplan-Meier method. The log-rank test was used to compare PFS between patients with oligo-residual disease and those with non-oligo residual disease. The end of the follow-up period was 31 December 2020. All categorical variables were analysed using the chi-square test. Potential predictive factors for oligo-residual disease were assessed using univariate and multivariate analyses with a logistic regression model adjusted for patient characteristics at baseline. Potential predictive factors for PD limited to the residual sites were assessed using univariate and multivariate analyses with a logistic regression model adjusted for patient characteristics at 3 months from the start of EGFR-TKI therapy. For the univariate analyses, the covariates included age (≥ 75 vs. < 75 years), sex, smoking status, ECOG-PS (0 – 1 vs. ≥ 2), histology (adenocarcinoma vs. non-adenocarcinoma), *EGFR* mutation (exon 19 deletions / L858R point mutations vs. others), residual central nervous system (CNS) metastases (yes vs. no), number of residual metastatic organs (0 – 1 vs. ≥ 2), [25, 26] EGFR-TKI type (osimertinib vs. other EGFR-TKIs), and oligo-residual disease. Factors with univariate *P*-values of < 0.1, were subjected to multivariate analyses. Differences were considered statistically significant at *P*-values of < 0.05. All analyses were performed using STATA version 14.0 (Stata Corp., Texas, USA).

## Results

### Baseline patient characteristics

A total of 207 patients were included in this study. The median patient age was 68 years (34 – 88 years). The majority of the patients had adenocarcinoma (94.7%), ECOG-PS 0 – 1 (82.6%), and sensitising *EGFR* mutations (95.1%). Only 6% of patients had synchronous oligometastatic disease, and 87% of patients had 10 or more metastases at the start of EGFR-TKI treatment (Table 1).

**Table 1:** Patient Characteristics at baseline and 3 months from EGFR-TKI

Characteristics ***N*** = 207 (%)	Baseline	3 months from EGFR-TKI		
	Overall N = 207	Oligo-residual n = 66 (%)	Non-Oligo ***n*** = 141 (%)	***P***
**Median age (range)**	68 (34-88)	67 (41-87)	68 (34-88)	0.478
**Sex**				
**Male**	77 (37)	22 (33)	55 (39)	0.431
**Female**	130 (63)	44 (67)	86 (61)	
**ECOG-PS**				
**0**	43 (21)	28 (42)	34 (24)	**0.021**
**1**	128 (62)	36 (55)	90 (64)	
**2**	26 (12)	2 (3)	15 (11)	
**3**	10 (5)	0	2 (1)	
**Smoking status**				
**Ever**	109 (53)	34 (52)	64 (45)	0.411
**Never**	98 (47)	32 (48)	77 (55)	
**Histology**				
**Adenocarcinoma**	196 (95)	61 (92)	135 (96)	0.321
**Non-Adenocarcinoma**	11(5)	5 (8)	6 (4)	
**Type of EGFR mutation**				
**Del 19**	124 (60)	33 (50)	91 (65)	0.391
**L858R**	73 (35)	30 (45)	43 (30)	
**Other**	10 (5)	3 (5)	7 (5)	
**Site of metastatic organs**				
**Pleura**	122 (59)	0	92 (65)	**< 0.001**
**Pulmonary**	114 (55)	17 (26)	63 (45)	**0.009**
**Bone**	95 (46)	12 (18)	62 (44)	**< 0.001**
**Brain**	71 (34)	6 (9)	32 (23)	**0.018**
**Liver**	36 (17)	7 (11)	30 (21)	**0.062**
**Adrenal grand**	24 (12)	5 (8)	19 (14)	**0.217**
**Extra-thoracic lymph node**	23 (11)	4 (6)	12 (9)	**0.538**
**Others**	13 (6)	2 (3)	10 (7)	**0.244**
**Number of lesions**				
**1**	0	17 (8)	0	
**2**	0	19 (9)	0	
**3**	3 (1)	20 (10)	0	
**4**	10 (5)	10 (5)	0	
**5**	5 (3)	0	2 (1)	
**6**	5 (3)	0	0	
**7**	1 (0)	0	2 (1)	
**8**	3 (1)	0	1 (0)	
**9**	0	0	0	
**≥10**	180 (87)	0	136 (66)	
**Number of metastatic organs**				
**0**	0	15 (22)	0	
**1**	63 (31)	39 (59)	60 (43)	
**2**	62 (30)	11 (17)	33 (23)	
**3**	46 (22)	1 (2)	20 (14)	
**4**	24 (12)	0	20 (14)	
**5**	5 (2)	0	3 (2)	
**6**	7 (3)	0	5 (4)	
**EGFR-TKI treatment**				
**Gefitinib**	96 (46)	30 (45)	66 (47)	**0.132**
**Erlotinib**	49 (24)	11 (17)	38 (27)	
**Afatinib**	24 (12)	8 (22)	16 (11)	
**Osimertinib**	38 (18)	17 (26)	21 (15)	

*ECOG* Eastern Cooperative Oncology Group, *PS* performance status, *EGFR* epidermal growth factor receptor, *TKI* tyrosine kinase inhibitor, *CNS* central nervous system, *DEL 19* exon 19 deletions, *L858R* L858R point mutations

### Oligo-residual disease

Three months after the start of EGFR-TKI treatment, 32% (*n* = 66) had oligo-residual disease. All 13 patients (100%) with synchronous oligometastatic disease at baseline had oligo-residual disease. Out of the 194 patients with non-synchronous oligometastatic disease at baseline, 53 patients (27%) showed oligo-residual disease at 3 months (Table 1).

Baseline clinical factors were investigated to identify the predictive factors of oligo-residual disease. The univariate logistic regression analysis demonstrated that ECOG-PS 0 – 1 (Odds ratio [OR] = 6.41, 95% confidence interval (CI) [1.89 – 21.77], *P* = 0.003) and the presence of only one metastatic organ (OR = 4.10, 95% CI [2.18 – 7.72], *P* < 0.001) were predictive factors for oligo-residual disease. The multivariate logistic regression analysis demonstrated that ECOG-PS 0 – 1 (OR = 4.98, 95% CI [1.41 – 17.6], *P* = 0.013), the presence of only one metastatic organ (OR = 3.59, 95% CI [1.86 – 6.93], *P* < 0.001) and treatment with osimertinib (OR = 2.33, 95% CI [1.06 – 5.13], *P* = 0.034) were independent predictive factors of oligo-residual disease (Table 2).

**Table 2:** Predictive factors of Oligo-residual disease using a logistic regression model adjusted for baseline patient characteristics

Covariates N = 207	Univariate analysis			Multivariate analysis		
	OR	95% CI	***P***-value	OR	95% CI	***P***-value
**Age (< 75 years vs ≥ 75 years)**	1.74	0.87-3.45	0.114			
**Sex (male vs female)**	1.29	0.69-2.36	0.432			
**ECOG performance status score (0-1 vs ≥ 2)**	**6.41**	**1.89-21.77**	**0.003**	**4.98**	**1.41-17.6**	**0.013**
**Smoking status (ever vs never)**	1.28	0.71-2.29	0.411			
**Histology (adeno vs non-adeno)**	0.54	0.15-1.84	0.327			
**EGFR mutation (del19/L858R vs Others)**	0.64	0.23-1.77	0.394			
**No baseline CNS metastases**	1.00	0.53-1.85	0.989			
**Baseline number of metastatic organ (1 vs ≥ 2)**	**4.10**	**2.18-7.72**	**< 0.001**	**3.59**	**1.86-6.93**	**< 0.001**
**Treatment with Osimertinib**	1.98	0.96-4.07	0.063	**2.33**	**1.06-5.13**	**0.034**

Significant *P*-values are shown in bold type. *OR* odds ratio, *CI* confidence interval, *ECOG* Eastern Cooperative Oncology Group, *adeno* adenocarcinoma, *EGFR* epidermal growth factor receptor, *DEL 19* exon 19 deletions, *L858R* L858R point mutations, *CNS* central nervous system

### Comparison between the oligo-residual and non-oligo-residual disease

The clinical characteristics of patients with oligo-residual disease and those with non-oligo-residual disease are summarised in Table 1. The distribution of ECOG-PS differed significantly between these two groups (*P* = 0.021). The predominant metastatic organs also differed significantly, as pleura, pulmonary, bone, and brain metastases were more frequently observed in patients with non-oligo-residual disease than in those with oligo-residual disease.

**Fig. 3:**
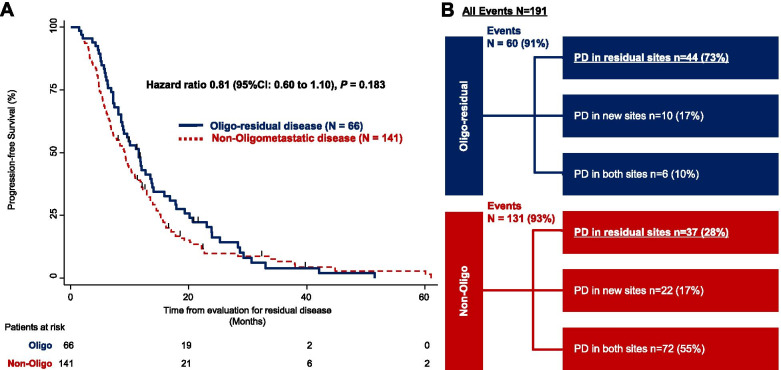
**A** Kaplan-Meier curves for progression-free survival of oligo-residual and non-oligo-residual disease. The *P*-value was calculated using the log-rank test. The small vertical lines on the curve indicate patients who were censored. **B** Pattern of progressive disease. EGFR, epidermal growth factor receptor; PD, progressive disease; TKI, tyrosine kinase inhibitor

The median follow-up time for PFS was 16 months. The median PFS was 11.3 months (95% CI [8.7 – 13.7]) in patients with oligo-residual disease and 9.1 months (95% CI [7.0 – 10.2]) in patients with non-oligo-residual disease. There was no significant difference in PFS between patients with oligo-residual disease and those with non-oligo-residual disease (hazard ratio = 0.81, 95% CI [0.60 – 1.10], *P* = 0.183) (Fig. 3).

### Pattern of PD

A total of 191 patients (92%) had PD, 60 of whom (91%) had oligo-residual disease, and 131 (93%) with non-oligo-residual disease. PD limited to residual sites was observed in 44 patients (73%) with oligo-residual disease and 37 patients (28%) with non-oligo-residual disease. The proportion of PD in the residual sites alone was significantly higher in patients with oligo-residual disease than in those with non-oligo-residual disease (*P* < 0.001) (Fig. 3).

The clinical factors at 3 months from the start of EGFR-TKI treatment were investigated to identify the predictive factors of PD limited to residual sites. The univariate logistic regression analysis demonstrated that ECOG PS 0 – 1 (OR = 2.96, 95% CI [1.26 – 6.94], *P* = 0.012), the lack of CNS metastases (OR = 4.15, 95% CI [1.72 – 10.00], *P* = 0.002), residual metastatic organ number of 0 – 1 (OR = 2.53, 95% CI [1.39 – 4.61], *P* = 0.002), oligo-residual disease (OR = 6.98, 95% CI [3.51 – 13.24], *P* < 0.001), and treatment with osimertinib were associated with PD limited to residual disease sites (OR = 3.13, 95% CI [1.27 – 7.74], *P* = 0.013). The multivariate logistic regression analysis demonstrated that the lack of CNS metastases (OR = 3.07, 95% CI [1.10 – 8.52], *P* = 0.032), oligo-residual disease (OR = 5.43, 95% CI [2.57 – 11.46], *P* < 0.001), and treatment with osimertinib (OR = 3.24, 95% CI [1.13 – 9.25], *P* = 0.028) were independent predictors of PD limited to residual disease sites (Table 3).

**Table 3:** Predictive factors of PD limited to residual sites using a logistic regression model adjusted for patient characteristics

Covariates ***N*** = 191	Univariate analysis			Multivariate analysis		
	OR	95% CI	***P***-value	OR	95% CI	***P***-value
**Age (< 75 vs ≥ 75 years)**	1.14	0.60-2.17	0.669			
**Sex (male vs female)**	1.05	0.58-1.90	0.853			
**ECOG performance status score (0-1 vs ≥ 2)**	**2.96**	**1.26-6.94**	**0.012**	1.77	0.67-4.66	0.249
**Smoking status (ever vs never)**	1.65	0.92-2.95	0.088	1.41	0.72-2.74	0.315
**Histology (adeno vs non-adeno)**	1.11	0.30-4.07	0.874			
**EGFR mutation (del19/L858R vs Others)**	2.60	0.69-9.76	0.157			
**No residual CNS metastases**	**4.15**	**1.72-10.00**	**0.002**	**3.07**	**1.10-8.52**	**0.032**
**Number of residual metastatic organ (0-1 vs ≥ 2)**	**2.53**	**1.39-4.61**	**0.002**	1.02	0.48-2.14	0.951
**Oligo-residual disease**	**6.98**	**3.51-13.24**	**< 0.001**	**5.43**	**2.57-11.46**	**< 0.001**
**Treatment with Osimertinib**	**3.13**	**1.27-7.74**	**0.013**	**3.24**	**1.13-9.25**	**0.028**

Significant *P*-values are shown in bold type. *OR* odds ratio, *CI* confidence interval, *ECOG* Eastern Cooperative Oncology Group, *adeno* adenocarcinoma, *EGFR* epidermal growth factor receptor, *CNS* central nervous system m, *del 19* exon 19 deletions, *L858R* L858R point mutations

Additionally, we investigated the clinical factors that were predictors of PD limited to the residual site prior to EGFR-TKI treatment initiation. Multivariate logistic regression analysis demonstrated that ECOG PS 0 – 1 (OR = 2.55, 95% CI [1.07 – 4.05], *P* = 0.012), the absence of CNS metastases (OR = 1.95, 95% CI [1.00-3.80], *P* = 0.049), and treatment with osimertinib were associated with PD limited to residual sites (OR = 3.33, 95% CI [1.22 – 9.06], *P* = 0.018). However, there was no significant association between the presence of oligometastatic disease or the number of organs involved in metastasis, and PD limited to residual sites (Supplementary Table 1).

## Discussion

This is the first study that assesses the pattern of PD in patients with *EGFR*-mutated NSCLC using the residual disease evaluation at 3 months after the start of EGFR-TKI treatment, including first-, second-, and third-generation EGFR-TKIs. Our study found that, after adjusting for other clinical factors, oligo-residual disease was an independent predictive factor of PD limited to the residual sites in patients with *EGFR*-mutated NSCLC. Analysing the pattern of PD after EGFR-TKI treatment might be essential to provide the rationale for LAT of all disease sites in patients with *EGFR*-mutated NSCLC, as well as in NSCLC patients without *EGFR* mutations [16, 18]. Our study also showed that more than 90% of patients had PD after EGFR-TKI. Acquired resistance remains a significant obstacle to achieving a durable response, even if the patients had oligo-residual disease response after EGFR-TKI treatment. Based on these data, adding consolidative LAT to all sites of residual disease at the time of response to EGFR-TKIs could potentially delay the time to progression or even improve survival outcomes in patients with oligo-residual disease.

The results of our study show that the majority of patients with oligo-residual disease have PD limited to residual sites after EGFR-TKI treatment. Preclinical studies have demonstrated that synchronous oligometastatic disease does not metastasise to other sites [27, 28]. EGFR-TKI treatment might have caused *EGFR*-mutated polymetastatic disease to become oligometastatic, which was defined as oligo-residual disease. Furthermore, our study showed that only 6% of patients had synchronous oligometastatic disease before treatment, which increased to 32% after treatment. These findings suggest that EGFR-TKI treatment for 3 months increased the number of patients eligible for LAT more than fivefold.

Limited to the small number of cases in our study, treatment with osimertinib was found to be an independent predictor of oligo-residual disease and PD limited to residual sites. No previous study has demonstrated the differences in PD patterns between osimertinib and other EGFR-TKIs. The FLAURA study showed that osimertinib was more effective than first-generation EGFR-TKIs, and suggested that the incidence of CNS relapses was lower in patients treated with osimertinib than in those treated with first-generation EGFR-TKIs (19% vs. 43%) [4, 5]. A recent retrospective study showed that osimertinib might delay the development of CNS metastasis compared to first-generation EGFR-TKIs [29, 30]. Intriguingly, the results of our study suggest that more effective EGFR-TKI treatment for CNS metastases may result in oligo-residual disease and PD limited to the residual sites. Additionally, the lack of residual CNS metastases at 3 months after the start of EGFR-TKI treatment was found to be an independent predictive factor of PD limited to residual sites in our study.

Previous studies have suggested that patients with brain metastases have a higher risk of developing newer brain metastases and have a poorer prognosis [31]. ECOG PS 0 – 1 and having one metastatic organ at baseline were independent predictive factors of oligo-residual disease in patients with *EGFR*-mutated NSCLC. Previous studies have already shown that both were prognostic factors in *EGFR*-mutated NSCLC patients receiving EGFR-TKIs [26, 32].

There were some limitations to our study. Our analysis was limited by its retrospective nature and the inability to account for unknown confounders. A relatively small sample size has the potential to affect its statistical power. This study was conducted in a cohort from a single institution and was not validated independently. Moreover, the present study could not provide data on the molecular basis for the association of osimertinib use with the occurrence of residual disease and PD limited to residual sites. Future research should focus on the molecular mechanisms underlying these associations. Patients with few baseline metastases (oligometastasis) could not be included in the multivariate analysis because they all developed oligo-residual disease after EGFR-TKI treatment induction. Although not all patients had undergone comprehensive imaging at the time of disease progression, the patients were categorised based on all available imaging findings. Furthermore, this study included only patients who were able to continue EGFR-TKI therapy for 3 months, which may have resulted in selection bias. Additionally, since first-line treatment with osimertinib was approved in Japan in August 2018, the median follow-up period for these patients was 15.8 months, shorter than the 37.8 months for patients treated with first- and second-generation EGFR-TKIs. Thus, bias due to differences in follow-up periods cannot be ruled out. A randomised phase II study of osimertinib with or without local consolidation therapy for stage IV NSCLC (ClinicalTrials.gov identifier: NCT03410043) is currently ongoing. The results of a prospective randomised study could confirm our hypothesis. Moreover, from a strictly scientific perspective, counting the number of residual lesions might be a crude surrogate marker of oligo-residual disease. Evaluation of the number of alleles with *EGFR* mutations in circulating cell-free DNA during EGFR-TKI treatment has been applied in several studies. Recent studies have suggested that minimal residual disease can detect changes in circulating cell-free DNA [33, 34, 35]. Thus, this has the potential to become a new method of identifying true oligo-residual disease [36].

## Conclusions

In conclusion, our study revealed that oligo-residual disease was significantly associated with PD limited to residual sites after EGFR-TKI treatment in patients with *EGFR*-mutated NSCLC. These results provide a rationale for LAT of all disease sites in patients with *EGFR*-mutated NSCLC and oligo-residual disease. Future studies should focus on the development of treatment strategies, including LAT, in patients with oligo-residual disease during treatment with EGFR-TKI.

## Supplementary Information

**Additional file 1: Supplementary Table 1**. Predictive factors of PD limited to residual sites using a logistic regression model adjusted for patient characteristics at baseline.

## Data Availability

The datasets used and/or analysed during the current study are available from the corresponding author on reasonable request.

## Abbreviations

CNS: Central nervous system
CI: Confidence interval
ECOG-PS: Eastern Cooperative Oncology Group performance status
EGFR-TKI: Epidermal growth factor receptor-tyrosine kinase inhibitor
LAT: Local ablative therapy
NSCLC: Non-small-cell lung cancer
OR: Odds ratio
OS: Overall survival
PFS: Progression-free survival
PD: Progressive disease
