# Supplementary material for: Association between oligo-residual disease and patterns of failure during EGFR-TKI treatment in EGFR-mutated non-small cell lung cancer: a retrospective study
Source: BMC Cancer. 2021 Nov 19;21:1247. doi: 10.1186/s12885-021-08983-2 (PMC8605535; doi:10.1186/s12885-021-08983-2)
Supplement: Supplementary file 1 — Additional file 1: Supplementary Table 1. Predictive factors of PD limited to residual sites using a logistic regression model adjusted for patient characteristics at baseline. [file 12885_2021_8983_MOESM1_ESM.docx]

| **Supplementary Table1.  Predictive factors of PD limited to residual sites using a logistic regression model adjusted for patient characteristics at baseline** | | | | | | |
| --- | --- | --- | --- | --- | --- | --- |
| **Covariates**  **N=191** | **Univariate analysis** | | | **Multivariate analysis** | | |
|  | **OR** | **95% CI** | ***P*-value** | **OR** | **95% CI** | ***P*-value** |
| **Age (< 75 vs ≥ 75 years)** | 1.29 | 0.68-2.42 | 0.426 |  |  |  |
| **Sex (male vs female)** | 1.05 | 0.58-1.90 | 0.853 |  |  |  |
| **ECOG performance status score (0-1 vs ≥2)** | **2.77** | **1.24-6.15** | **0.012** | **2.55** | **1.07-4.05** | **0.033** |
| **Smoking status (ever vs never)** | 1.65 | 0.92-2.95 | 0.088 | 1.40 | 0.76-2.59 | 0.278 |
| **Histology (adeno vs non-adeno)** | 1.11 | 0.30-4.07 | 0.874 |  |  |  |
| **EGFR mutation (del19/L858R vs Others)** | 2.60 | 0.69-9.76 | 0.157 |  |  |  |
| **No CNS metastases** | **2.27** | **1.23-4.34** | **0.009** | **1.95** | **1.00-3.80** | **0.049** |
| **Number of metastatic organ (0-1 vs ≥2)** | 1.84 | 0.98-3.47 | 0.057 | 1.21 | 0.59-2.49 | 0.586 |
| **Oligometastatic disease** | 3.46 | 0.90-13.2 | 0.069 | 2.08 | 0.50-8.61 | 0.311 |
| **Treatment with Osimertinib** | **2.98** | **1.27-7.74** | **0.013** | **3.33** | **1.22-9.06** | **0.018** |

Significant *P*-values are shown in bold type. OR, odds ratio; CI, confidence interval; ECOG, Eastern Cooperative Oncology Group; adeno, adenocarcinoma; EGFR; epidermal growth factor receptor; CNS, central nervous system m; del 19, exon 19 deletions; L858R, L858R point mutations.
